# Malformation anorectale avec fistule recto-uretro-bulbaire prise en charge tardivement: à propos d'un cas

**DOI:** 10.11604/pamj.2019.33.223.17810

**Published:** 2019-07-18

**Authors:** Tresor Kibangula Kasanga, Jeef Bukasa Misenga, Manix Ilunga Banza, Nathalie Dinganga Kapessa, Tshiband Mosh Bilond, Prince Muteba Katambwa, Dimitri Kanyanda Nafatalewa, Stephanne Ilunga Muka Ngala, Tshibwid Azf, Papy Mukimba Ngabunda, Didier Tshibangu Mujinga

**Affiliations:** 1 Département de Chirurgie, Faculté de Médecine, Clinique Universitaire de Lubumbashi, Université de Lubumbashi, Province du Haut-Katanga, République Démocratique du Congo

**Keywords:** Malformation anorectale, fistule recto-urethro-bulbaire, filière rectale, Anorectal malformations, rectouretrobulbar fistula, rectal canal

## Abstract

Les malformations ano-rectales (MAR) sont un ensemble hétérogène d'anomalies de la mise en place de la filière rectale. Son incidence reste faible en général et se trouve un peu plus élevée dans certains pays en développement. Les garçons sont plus atteints que les filles et la fistule recto-bulbaire associée à une atrésie du canal anal demeure la forme la plus fréquente chez eux. Nous présentons un nourrisson de sexe masculin âgé de 10 mois, dont la mère habite dans une région minière et s'est plainte de fecalurie depuis la naissance de son enfant. L'examen physique a révélé la présence d'une empreinte à 1 cm au-dessous du croisement du raphé médian et de la ligne bi-ischiatique. Les examens paracliniques n'ont montré aucune autre malformation associée. La prise en charge a consisté en une ano-rectoplastie par double abord, abdominal et périnéal, en réalisant la technique d'abaissement abdominopérinéal. En période post-opératoire, une antibiothérapie et une analgésie intraveineuses ont été instaurées, de même que les dilatations, lesquelles ont été poursuivies après la sortie du patient 2 semaines après l'intervention. Aucune complication n'a été observée et les suites ont été favorables.

## Abstract (en)

Ano-rectale malformations (ARM) are a spectrum of heterogeneous abnormalities in the development of the rectal canal. Its incidence is usually low and it is a little higher in some developing countries. Boys are more affected than girls and rectobulbar fistula associated with atresia of the anal canal is the most frequent disorder among them. We report the case of a 10-months old infant of male sex, whose mother lived in a mining area and had been complaining of fecaluria since the birth of her child. Physical examination showed fingerprint 1 cm below the intersection of the median raphe and the bi-ischiatic line. Paraclinical examinations showed no other associated malformation. Patient's management was based on anorectoplasty through abdominal and perineal approach with lower abdominoperineal reconstruction. In the post-operative period, the patient received antibiotic treatment and intravenous analgesia as well as dilations, which continued after discharge 2 weeks after surgery. No complications were observed and outcome was favorable.

## Introduction

Les malformations ano-rectales (MAR) regroupent un large spectre d'anomalies congénitales allant de l'imperforation anale à la persistance du cloaque [1]. Elles concernent toute anomalie de mise en place de la filière rectale survenant entre la sixième et la dixième semaine de développement embryonnaire. Les MAR couvrent un large spectre des malformations au pronostic fonctionnel hétérogène s'associant dans 60% des cas à d'autres malformations [2]. Ces MAR sont des maladies rares, dont l'incidence varie de 1/2500 à 1/5000 naissances mais celle-ci pourrait être plus élevée dans certains pays en développement [3-5]. Ces différences d'incidences sont liées aux variations géographiques, ethniques et génétiques [6]. Les garçons sont légèrement plus touchés [7-9], le sexe ratio est de 1,6/1. Dans une étude menée à l'Est de la RDC a trouvé que les MAR représentaient 21,1% de toutes les malformations alors qu'à Lubumbashi, Lubala a trouvé une prévalence de 10,6% [10]. La prise en charge a un double objectif: refaire l'anatomie rectale/anale et génito-urinaire et du point de vue fonctionnel, de maintenir la continence urino-fécale [11]. Pour cela, plusieurs interventions médicales et chirurgicales peuvent être nécessaires [11]. Cette prise en charge doit se faire en période néonatale car d'elle dépend le devenir immédiat du nouveau-né [12]. Cependant, des cas de prise en charge tardive ont été rapportés et ce, essentiellement dans les pays en développement [13,14]. Cet article présent un cas rare des malformations anorectales (imperforation anale avec fistule recto-uretrobulbaire prise en charge tardivement chez un nourrisson.

## Patient et observation

**Figure 1:**
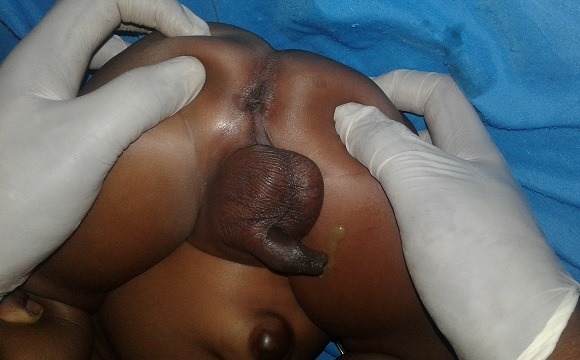
Inspection de l'imperforation anale + fécalurie

**Figure 2:**
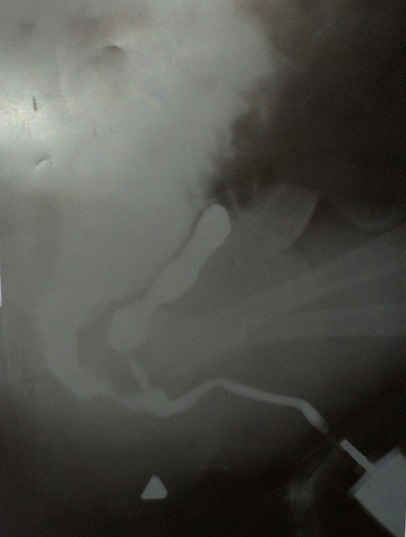
Fistulographie avec produit de contraste

**Figure 3:**
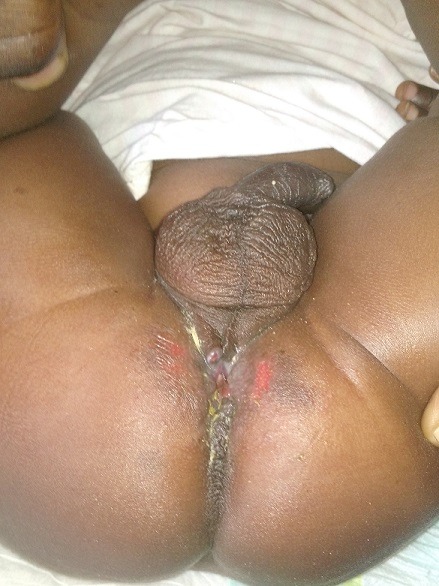
Image au 21^ème^ jour post opératoire

Nous rapportons le cas d'un nourrisson de 10 mois de sexe masculin, né à terme, qui avait pesé à la naissance 2300g admis au service de chirurgie des cliniques universitaires de Lubumbashi en date du 16/9/2018 pour la non émission des selles par l'anus et l'émission des selles par le méat urétral depuis sa naissance. Dans ses antécédents, nous n'avons pas trouvé la notion d'infection urogénitale pendant le 1^er^ trimestre chez la mère, ni la notion de prise des médicaments tératogène dans cette période. Signalons aussi que la mère ne prenait pas l'alcool et ne fumer pas pendant la même période, et vivait dans une région minière avant et pendant la grossesse. Cependant, on ne signale aucune malformation congénitale visible dans la famille. Au complément d'anamnèse, la mère signale l'émission des urines mélangées aux matières fécales, de coloration jaune verdâtre depuis la naissance par le méat urétral (fécalurie) et l'absence d'émission des selles par l'anus. Pas de notion de vomissement, pas de notion de ballonnement abdominal, ni de fièvre. À l'examen physique, son état général était bon et les signes vitaux étaient dans les normes physiologiques. À l'examen locorégional, le patient en position de taille, nous avions noté la présence de l'empreinte anale (Figure 1). Une fistulographie a montré une communication anormale entre l'urètre bulbaire et le rectum (Figure 2) qui, par ailleurs, était la forme classique par rapport à la classification Krickenbeck. La radiographie du rachis incidence face et profil avait montré une colonne vertébrale bien formée. Le sondage vésical a noté l'issu des matières fécales mélangées aux urines faisant suspecter une forme rare d'une malformation ano-rectale et fistule recto-urétrale. Des bilans malformatif et préopératoire ont été réalisés et ont révélés ce qui suit: Echographie abdomino-pelvienne a montré les reins avec bonne différentiation corticomedullaire; à la biologie l'urée 16 mg%; créatinine 0,7% mg; temps de saignement 3 secondes; temps coagulation 6 secondes, hémoglobine 14%, hématocrite 42%. Nous avons réalisé une anorectoplastie avec fermeture de la fistule par voie périnéo-abdominale. Le premier temps, périnéal a été une incision cruciforme centrée sur l'emprunt anale. Une dissection des structures profondes a été faite jusqu'à la mise en évidence du cul de sac rectal. Le second temps, abdominal a été une laparotomie médiane sous-ombilico-sus pubienne qui a révélé un côlon sigmoïde très dilaté avec son méso intact et présence du trajet fistuleux entre l'urètre bulbaire et le rectum. Ensuite fermeture de la paroi abdominale. La sonde urinaire numéro 6 placé en préopératoire a été perçue dans le rectum ensuite retirée. Au niveau de l'incision périnéale, la dissection du tissu cellulaire sous-cutané a montré le trajet fistuleux recto-bulbaire. Une ligature-section du trajet fistuleux a été réalisée. Nous avons replacé une nouvelle sonde urinaire de même calibre du méat urétral jusque dans la vessie. La descente du cul de sac rectal jusqu'à la région périnéale suivie de son ouverture et de la suture entéro-cutanée en deux plans en points séparés éversants ont été faites. En période postopératoire, le malade était pris en charge par une équipe mixte associant les réanimateurs et les chirurgiens. Il a bénéficié d'une antibiothérapie (Cefotaxime 3x500mg), un apport liquidien en perfusion (Sérum Physiologique, Sérum glucosé 5% et Sérum Ringer Latacte, au total 1,5 L pendant 24H), une analgésie par voie parentérale (Paracétamol infusion a raison de 2x500mg). La cicatrisation est survenue au 21 ^ème^ jour et nous avons envisagé sa sortie (Figure 3). Et au reste, continuer avec les séances de dilatations anales.

## Discussion

Les MAR regroupent un complexe hétérogène des anomalies intéressant l'anus et le rectum, tout comme les systèmes urinaire et génital dont le pronostic fonctionnel est proportionnel à la simplicité ou complexité des anomalies; les plus complexes étant difficile à prendre en charge et associant souvent d'autres anomalies [11,14]. L'étiologie reste floue; probablement multifactoriel. La cause génétique est à ne pas écarter [15]. La fièvre pendant le premier trimestre de la grossesse et les emplois responsables de l'exposition maternelle aux agents de nettoyage (les solvants) sont des facteurs qui ont été incriminés pour engendrer ces types de malformations [16]. L'incidence est autour de 1/5000 naissances, mais est reconnue pour certains auteurs [16] alors que pour d'autres, la prédominance est féminine comme étant plus élevée dans les milieux Africains [14]. Les garçons sont légèrement plus touchés [8,9,17], la forme la plus fréquente étant chez eux, l'atrésie anale associée à une fistule recto-urétrale; alors que chez les filles, il s'agit de l'atrésie associée à une fistule recto-vestibulaire [9]. Ces malformations sont en défaveur du sexe féminin [18]. Ceci rejoint notre observation; le patient étant du sexe masculin et ayant présenté une fistule recto-urétrale associée à une atrésie du canal anal. Approximativement 60% des patients ont une malformation associée [1]. Les incidences varient selon les études, mais les plus rencontrées sont génito-urinaires (40-50%), cardiovasculaires (30-35%), vertébrales (25-30%), gastro-intestinales (5-10%) et l'association VACTERL (vertebral, anal, cardiac, tracheal esophagal, fistula, renal, limb) (4-9%) [19]. Cela n'a pas été le cas chez notre patient qui n'avait aucune malformation visible.

Depuis la classification de Ladd et Gross de 1934, plusieurs autres ont été proposées et depuis 2005, c'est le système international de Krickenbeck qui est utilisé. Il regroupe les formes cliniques majeures et les variantes régionales/locales [5]. La forme de notre patient était classée parmi les formes cliniques majeures, appelées ainsi car étant les plus fréquentes. C'est fait habituellement à la naissance lors de l'examen physique. Le médecin remarque un anus non-ouvert ou placé anormalement. Dans les pays en développement, les principales raisons du retard de diagnostic sont le faible niveau intellectuel, la désinformation et la pauvreté [13]. Le retard de prise en charge de notre patient (10 mois de vie), illustre encore mieux le challenge que représente la prise en charge des MAR dans les milieux à faibles revenus. Dans ces pays en développement, le retard de diagnostic et le manque de chirurgiens pédiatres rendent complexe la prise en charge des MAR [14]. La PEC chirurgicale peut être réalisée avec ou sans colostomie préalable, selon la présence ou non d'une fistule périnéale ou vestibulaire, ou encore la présence ou pas d'une constipation chronique, dans les présentations tardives [11,13]. Elle consiste en une anorectoplastie sagittale, laquelle peut être faite par voie postérieure (la technique la plus utilisée actuellement) ou antérieure. L'abaissement abdominopérinéal est encore utilisé dans nos milieux [17]. Dans les soins postopératoires, les dilatations rapides, mais délicates sont réalisée à l'aide des bougies de Hegar, en commençant par le numéro 8, jusqu'au 14 [20]. L'abaissement abdominopérinéal a été la technique chirurgicale utilisée chez notre patient, suivi des dilatations à partir du 21^ème^ jours post opératoire. De nos jours, grâce à une meilleure compréhension de l'anatomie et à l'expérience dans la prise en charge, des meilleurs résultats sont obtenus [12]. Mais dans les pays à faibles revenus, les complications sont relativement fréquentes et la mortalité reste alarmante [14]. En ce qui concerne notre patient, nous n'avons pas eu de complication et le pronostic fonctionnel a cours terme a était bon.

## Conclusion

Les MAR dans leurs formes anatomiques de fistule recto-urétrale comporte intrinsèquement un risque élevé d'infection urinaire avec retentissement sur le haut appareil, surtout si le diagnostic est posé tardivement, d'où l'intérêt d'un diagnostic précoce anténatal, néonatal et de référer les cas dans un centre expert afin d'assurer une chirurgie d'excellente qualité. Le retard encore porté au diagnostic suggère une sensibilisation plus accrue des matrones, sages-femmes, obstétriciens et pédiatres, et une vulgarisation de l'inspection systématique du périnée de tout nouveau-né. Le résultat fonctionnel à terme résume tout l'enjeu de cette chirurgie qui doit conférer au patient une continence socialement acceptable.

## Conflits d’intérêts

Les auteurs ne déclarent aucun conflit d'intérêts.
